# Weighted Gene Coexpression Network Analysis Reveals Essential Genes and Pathways in Bipolar Disorder

**DOI:** 10.3389/fpsyt.2021.553305

**Published:** 2021-03-17

**Authors:** Zhen-Qing Zhang, Wei-Wei Wu, Jin-Dong Chen, Guang-Yin Zhang, Jing-Yu Lin, Yan-Kun Wu, Yu Zhang, Yun-Ai Su, Ji-Tao Li, Tian-Mei Si

**Affiliations:** ^1^Xiamen Xianyue Hospital, Xiamen, China; ^2^Peking University Sixth Hospital, Peking University Institute of Mental Health, Peking University, Beijing, China; ^3^Department of Psychosomatic Medicine, First Teaching Hospital of Tianjin University of Traditional Chinese Medicine, Tianjin, China; ^4^Institute of Mental Health, Hebei North University, Hebei, China

**Keywords:** bipolar disorders, coexpression modules, hub genes, WGCNA, pathway analysis

## Abstract

Bipolar disorder (BD) is a major and highly heritable mental illness with severe psychosocial impairment, but its etiology and pathogenesis remains unclear. This study aimed to identify the essential pathways and genes involved in BD using weighted gene coexpression network analysis (WGCNA), a bioinformatic method studying the relationships between genes and phenotypes. Using two available BD gene expression datasets (GSE5388, GSE5389), we constructed a gene coexpression network and identified modules related to BD. The analyses of Gene Ontology and Kyoto Encyclopedia of Genes and Genomes pathways were performed to explore functional enrichment of the candidate modules. A protein-protein interaction (PPI) network was further constructed to identify the potential hub genes. Ten coexpression modules were identified from the top 5,000 genes in 77 samples and three modules were significantly associated with BD, which were involved in several biological processes (e.g., the actin filament-based process) and pathways (e.g., MAPK signaling). Four genes (*NOTCH1, POMC, NGF*, and *DRD2*) were identified as candidate hub genes by PPI analysis and CytoHubba. Finally, we carried out validation analyses in a separate dataset, GSE12649, and verified *NOTCH1* as a hub gene and the involvement of several biological processes such as actin filament-based process and axon development. Taken together, our findings revealed several candidate pathways and genes (*NOTCH1*) in the pathogenesis of BD and call for further investigation for their potential research values in BD diagnosis and treatment.

## Introduction

Bipolar disorder (BD) is a chronic and recurrent severe mental disorder that affect about 1% global population (1). The disease is associated with high heritability, ranging from 70 to 90% (2), but its key genetic and neurobiological mechanisms are still not recognized. With the development of high-throughput sequencing technology, significant progress has been made in the genomics of BD. Recently, some systematic reviews and genome-wide association study (GWAS) findings have revealed more than 40 genes, including *ANK3, ERBB2, ODZ4, CACNA1C*, and *FADS* (3, 4). Pathway analyses of these genes showed that they were involved the regulation of insulin secretion, apoptosis, immunological response, neuroplasticity, HPA axis dysregulation, and the signal of endocannabinoid, etc., (5, 6). Recent RNA-sequencing studies also highlight dysregulation of neuroplasticity, circadian rhythms, as well as GTPase binding in BD (7). These findings provide clear evidence that BD is associated with an extensive polygenic genetic architecture (5), which calls for the network-level investigation to reveal the correspondence between risk genes and phenotypes. By combining public microarray data with bioinformatic analysis, we can gain an in-depth understanding of the molecular processes and pathogenesis of BD.

Weighted gene coexpression network analysis (WGCNA) is a bioinformatic method to study the relationships between genes and phenotypes. Recently, this technique has been widely applied to neurological and psychiatric disorders, including posttraumatic stress disorder, depression, schizophrenia, Alzheimer's disease, and Huntington's disease (8–13). Different from previous research methods focusing on individual genes, WGCNA transforms gene expression profiles into coexpression networks (modules). By examining modules of highly correlated genes, this method provides insights into the signaling networks that may be responsible for phenotypic traits of interest and the results may help identify the candidate biomarkers or therapeutic targets of many biological processes (14, 15).

The aim of this study is to reveal gene modules related to BD and to identify their functional pathways and potential hub genes. Using two available BD gene expression datasets (GSE5388, GSE5389), we constructed a gene coexpression network using WGCNA. For the modules related to BD, we then performed the Gene Ontology (GO) and Kyoto Encyclopedia of Genes and Genomes (KEGG) analyses to reveal the pathways enriched in these modules. In addition, a protein-protein interaction (PPI) network was constructed for the module of interest to identify candidate hub genes. Finally, we carried out validation analyses in a separate dataset GSE12649. These analyses based on gene coexpression profiles would shed light on the polygenic genetic architecture of BD and help to reveal the potential gene markers for BD diagnosis and treatment.

## Materials and Methods

### Microarray Data

Two gene expression datasets of BD patients, GSE5388, and GSE5389, were downloaded from the GEO database in May 2019 (https://www.ncbi.nlm.nih.gov/geo/). The GSE5388 dataset contained 61 samples (30 bipolar and 31 control subjects) from the dorsolateral prefrontal cortex (DLPFC). The GSE5389 dataset contained 21 samples (10 bipolar and 11 control subjects) from the orbitofrontal cortex (OFC). Both datasets were from the study of Ryan et al. (16) and based on the GPL96 platform ([HG-U133A] Affymetrix Human Genome U133A Array). The clinical data of these datasets, such as diagnosis, demographics (e.g., age, sex), and technical information (e.g., post-mortem interval, pH, RNA degradation, Batch), were also obtained. These two datasets were chosen based on prior evidence of involvement of DLPFC and OFC in BD (17). Ryan and colleagues found that gene expression changes in two regions were comparable (16), which suggests that the two datasets could be merged. Below we performed quality control analysis to further make sure that there are no batch effects between the two datasets.

### Data Preprocessing and Quality Control

The preprocessing was carried out separately for each dataset, including quality control and normalization, using the *affy* package in R (version 3.6.0; https://www.r-project.org/) (18). Demographic and technical variables were treated as covariates to control for their potential influence on the differences between bipolar and control subjects. The samples with standardized sample network connection Z-scores <-2 were excluded from further analysis. Five samples (GSM123204, GSM123205, GSM123206, GSM123214, and GSM123243) were defined as outliers and removed, resulting in 77 samples for final analysis. The chip scan date extracted from the metadata was used as an experimental batch for each dataset. The ComBat function of *sva* package in R was used to correct for batch effects (19). Annotations to the probes were performed using Ensembl gene IDs (v75; Feb 2014 data freeze) by the *biomaRt* package in R (20). A larger dataset was built by merging the two datasets following previous practics (12). The ComBat function was used to eliminate study batch effects if present (19).

### Weighted Gene Coexpression Network Analysis (WGCNA)

The WGCNA was performed on the average expression levels of the top 5,000 genes in 77 samples using the *WGCNA* package in R (14). The sample network connectivity was standardized by function scale according to the distance before WGCNA, which excluded the outlier samples connectivity <-5. The hierarchical clustering of samples was analyzed using the default method (hclust function), and no sample outliers were found. The soft thresholding power was then screened by the pickSoftThreshold function. Candidate powers from 1 to 20 were applied to test the mean connectivity degrees and the independence of modules. The soft thresholding power was selected if the *R*^2^ **≧** 0.8. To construct the WGCNA, the blockwiseModules function in R was used, and multiple parameters were defined. Here, the following parameters were used: power = 8, minModuleSize = 50, deepSplit = 1, networkType = “unsigned.” The module detection process was performed automatically by BlockwiseModules. Specifically, it can build a correlation network, create a cluster tree, and then merge nearby branches to form modules. A hierarchical clustering tree (dendrogram) was plotted to display hierarchical clustering. DissTOM (1-Topological Overlap Matrix) was calculated, and the relationships among all genes were visualized in R. Finally, the heatmap function in R was used to analyze the correlations between the modules.

### Relationship Analysis Between Coexpression Modules and BD

The module eigengene (ME) represents the first principal component in each module and therefore reflects the level of gene expression in the module (14). Pearson's correlation test was applied to assess the correlation between ME and BD and the heatmap package in R was used to visualize the correlations between modules and BD. Modules with significantly negative or positive correlations between ME and clinical traits were considered as candidate modules.

### GO and KEGG Analyses of Coexpression Modules for Bipolar Disorder

The Gene Ontology (GO) analysis, which includes the biological process (BP), cellular component (CC), and molecular function (MF) ontologies, is a standard method for gene functional annotation (21, 22). The Kyoto Encyclopedia of Genes and Genomes (KEGG) database, which stores gene metabolic pathways, is widely used to determine functional enrichment (23). Here, GO and KEGG analyses were performed for the genes in candidate modules in Metascape (http://metascape.org/gp/index.html#/main/step1), which is a reliable and widely used online software for omics-based research (24). The candidate modules were calculated with a *P*-value cutoff of 0.01, a min overlap of 3, and a min enrichment of 1.5.

### Protein-Protein Interaction Network Analysis for Selected Modules of Bipolar Disorder

The genes in candidate modules were mapped into the STRING database (Version 11.0, https://string-db.org/) for PPI network analysis. The Cytoscape software (v3.7.1) (25) was used for visualization, and the CytoHubba plug-in (http://hub.iis.sinica.edu.tw/cytohubba/) was applied to find hub genes in each module. To reduce potential errors caused by a complex biological network, it is necessary to use multiple methods to identify essential proteins (26). Hub genes were analyzed by CytoHubba using the following five methods: maximum neighborhood component, node connect degree, closeness, edge percolated component, and radiality (27–29).

### Validation Using Another Gene Expression Dataset of BD

The microarray dataset GSE12649, which was deposited by Iwamoto et al. (30), was used to validate our findings. This dataset contained 102 subjects from the prefrontal cortex (BA46), including 33 BD, 35 schizophrenia, and 34 control subjects. The BD and control samples were used for validation analysis. We carried out the same analyses as our main study, including data preprocessing, WGCNA, GO and KEGG analyses, PPI visualization, and hub genes screening. We validated our previous GO enrichment and KEGG pathway results in this dataset, focusing on top five GO enrichment results in each module and the important KEGG pathways. Finally, the hub genes of main and validation analyses were compared with each other to identify shared hub genes across datasets.

## Results

### Data Preprocessing and Quality Control

As shown in the boxplots and histograms, each microarray dataset indicated valid normalization and quality control for further analyses (Supplementary Figure 1). Bipolar and control status were not significantly associated with sex, age, pH, post-mortem interval (PMI), RNA degradation (RNAdeg), and Batch (*P* > 0.05). Five samples (GSM123204, GSM123205, GSM123206, GSM123214, and GSM123243) were defined as outliers and removed, resulting in 77 samples for final analysis. There were a total of 12,300 genes shared between the two datasets and no batch effects were observed (*P* > 0.05; see Supplementary Figure 2). The gene coexpression network was constructed by 5,000 genes with the highest average expression values (31).

### WGCNA to Identify Modules Critical to BD

**Figure 1:**
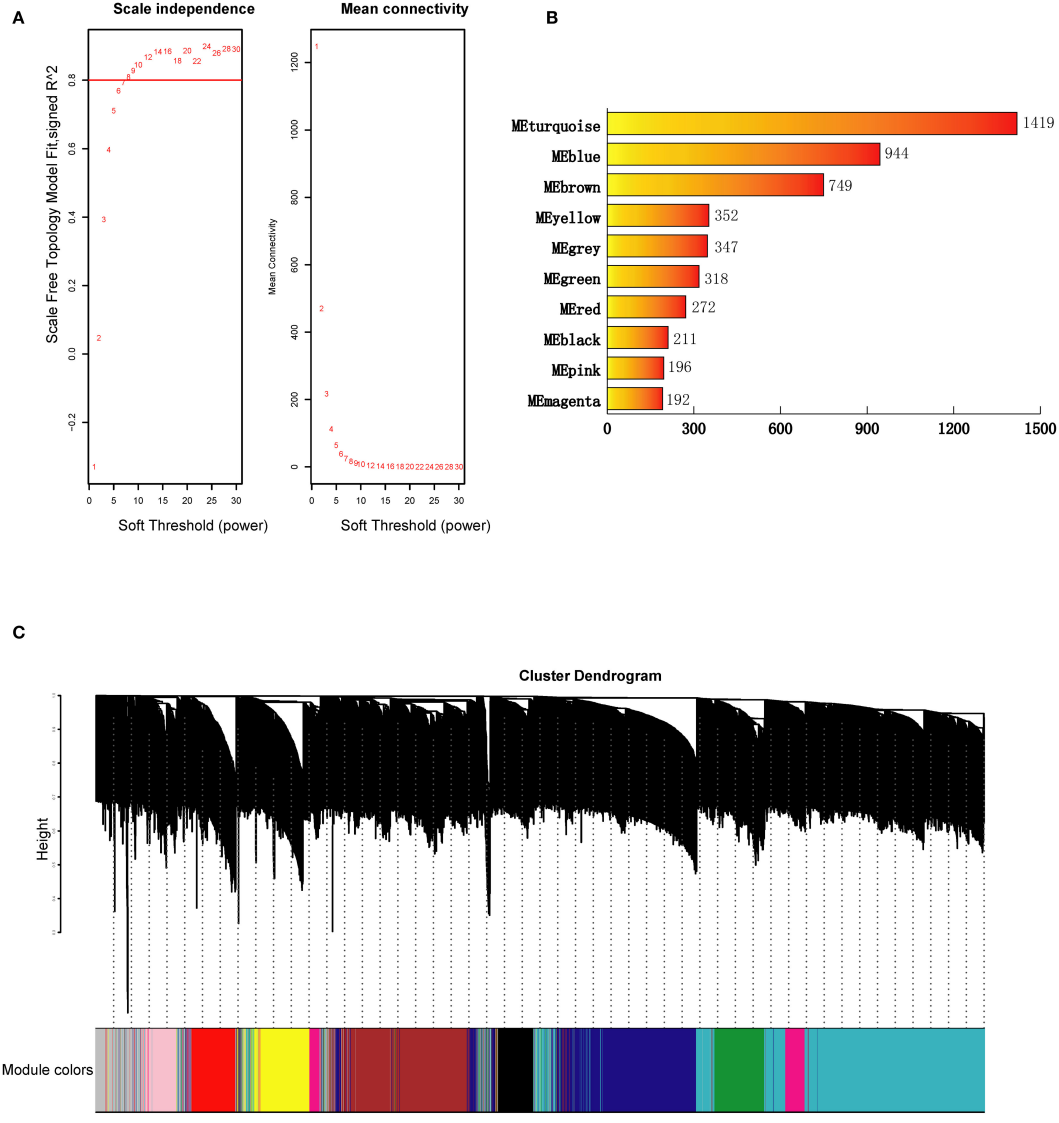
Construction of genes coexpression modules for BD. **(A)** Analysis of network topology for a set of soft-thresholding powers. **(B)** Number of genes in each coexpression module. **(C)** Construction of genes coexpression modules. Each color represents a module and each branch represents a gene. ME, module.

**Figure 2:**
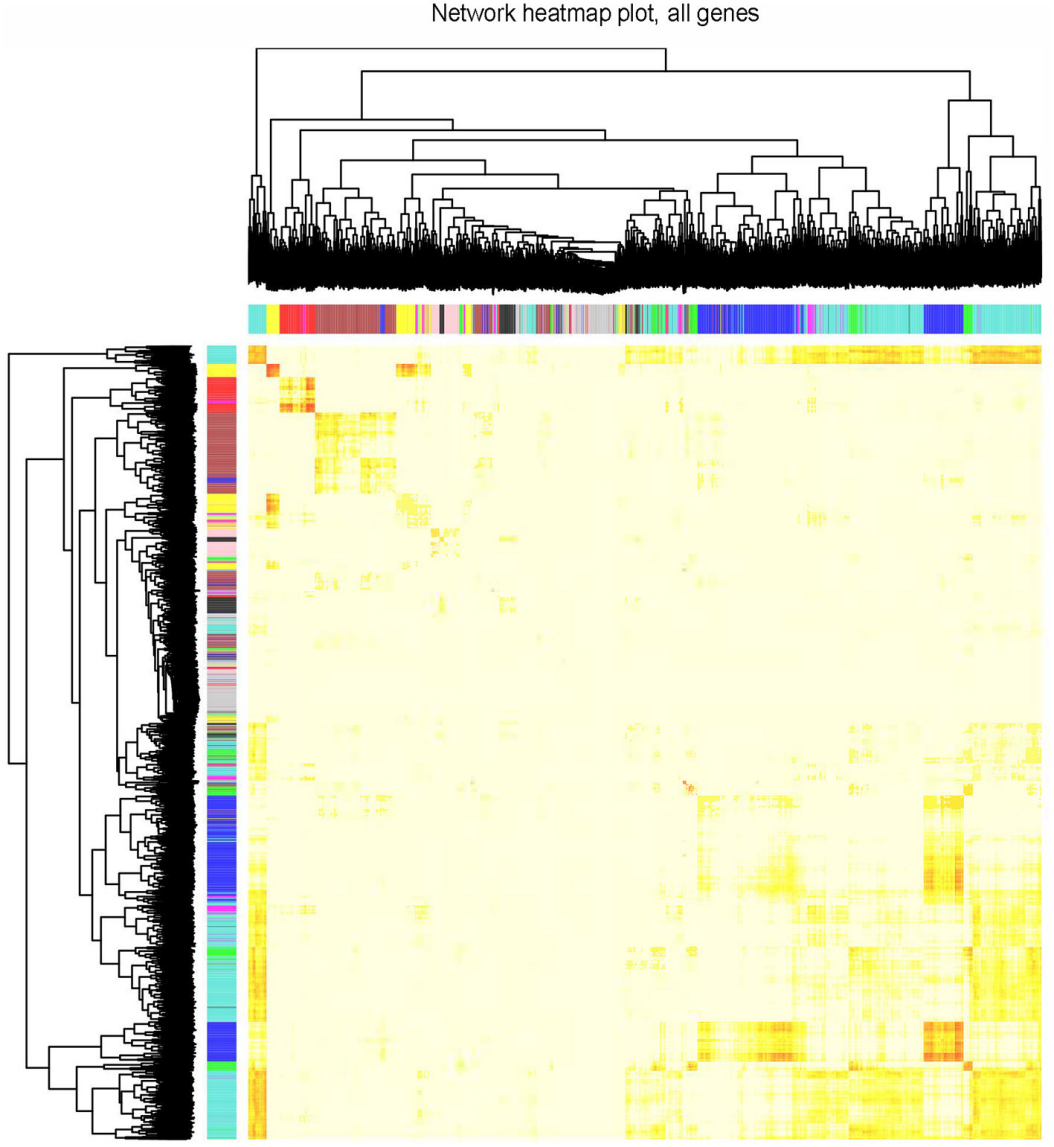
Visualization of TOM of co-expressed genes in different modules by a heat map. Light colors indicate low overlap and dark red indicates high overlap. The darker color blocks along the diagonal are coexpression modules.

The clustering results in Supplementary Figure 3 showed that all 77 samples were clustered well. With a soft-threshold power of 8 (Figure 1A), nine coexpression modules were identified, ranging in size from 192 to 1,419 genes (Figures 1B,C). Specifically, there were 1,419 genes in module 1 (MEturquoise), 944 genes in module 2 (MEblue), 749 genes in module 3 (MEbrown), 352 genes in module 4 (MEyellow), 318 genes in module 5 (MEgreen), 272 genes in module 6 (MEred), 211 genes in module 7 (MEblack), 196 genes in module 8 (MEpink), and 192 genes in module 9 (MEmagenta). In addition, 374 genes not assigned to any of the above modules were classified as a gene set Module 0 (MEgrey). The interactions among the ten modules are shown in Figure 2 and suggest that the modules were relatively independent.

**Figure 3:**
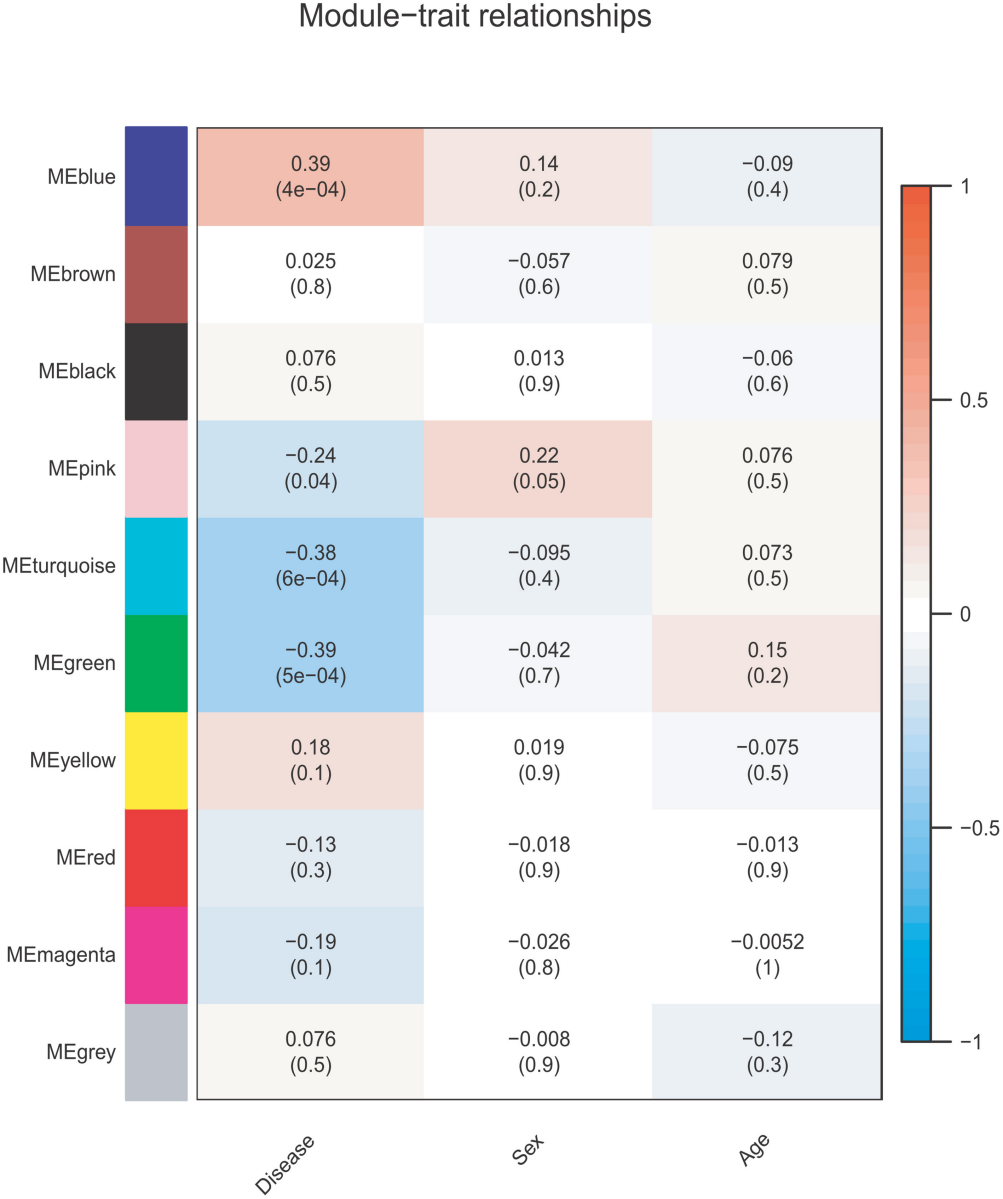
Module-trait relationship. Each row corresponds to a module eigengene, and each column corresponds to one feature. Each cell contains the corresponding correlation and *p-*value.

The correlations between the coexpression modules and clinical traits are shown in Figure 3. Three modules were significantly associated with BD status (Bonferroni-corrected *P* < 0.05). The MEblue module (*r* = 0.39, *P* = 4e-04, corrected *P* = 4e-03) showed a positive correlation, whereas the MEgreen (*r* = −0.39, *P* = 5e-04, corrected *P* = 5e-03), and MEturquoise (*r* = −0.38, *P* = 6e-04, corrected *P* = 6e-03) modules showed negative correlations. None of the 10 modules was significantly associated with sex or age (*Ps* > *0.05*).

### Gene Function and Annotation Enrichment (Functional Enrichment) Analysis of Key Modules

**Figure 4:**
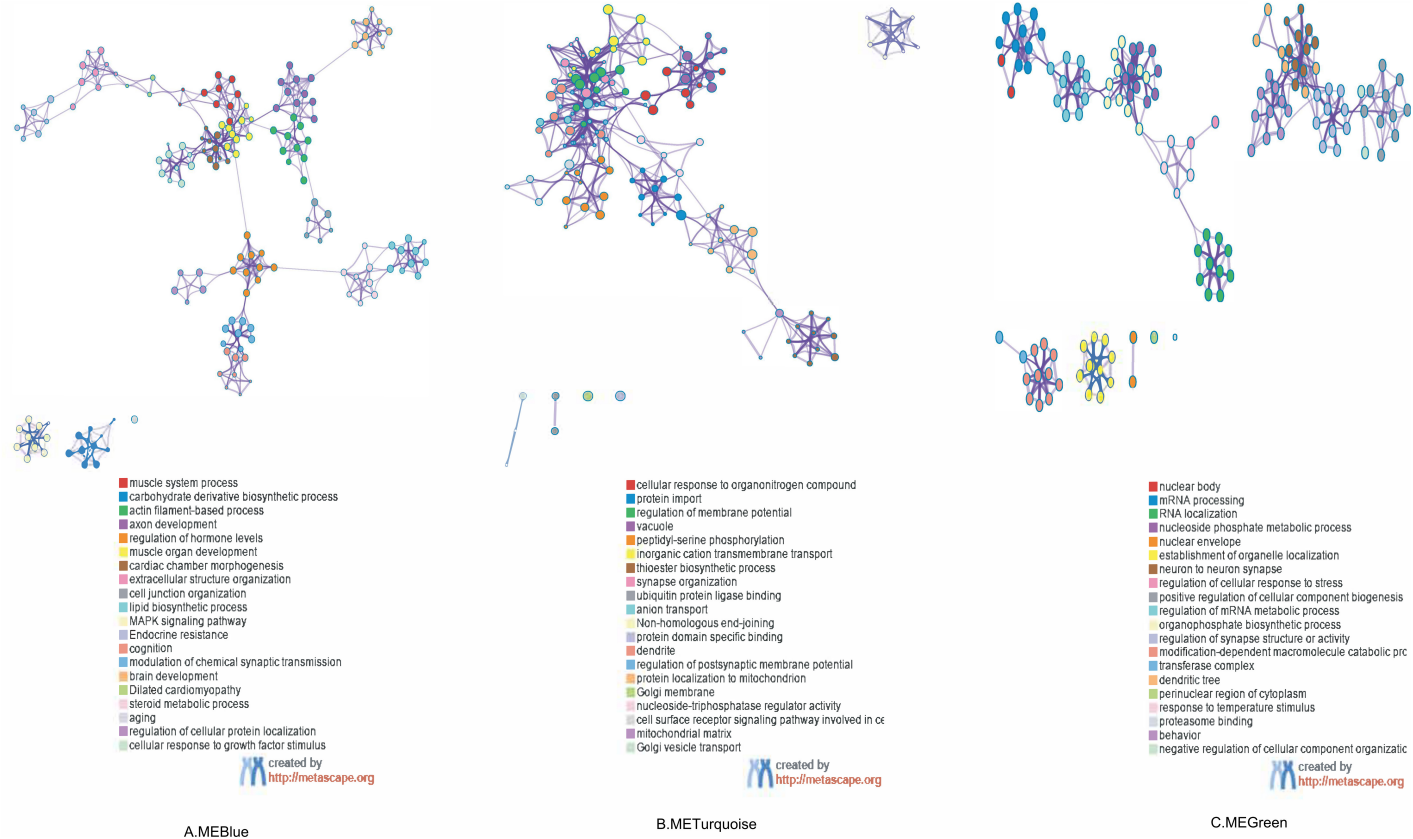
Functional enrichment analysis of three co-expressing network modules: enriched terms are represented by circle nodes, and nodes of the same color belong to the same cluster. **(A)** Colored by cluster ID in MEBlue, where nodes that share the same cluster ID are typically close to each other. **(B)** Colored by cluster ID in METurquoise. **(C)** Colored by cluster ID in MEGreen. ME, module.

As shown in Figure 4, the GO and KEGG pathway analyses were performed for the three key modules. The top five GO enrichment results showed that genes in MEblue were mainly enriched in GO:0003012 (muscle system process), GO:1901137 (carbohydrate derivative biosynthetic process), GO:0030029 (actin filament-based process), GO:0061564 (axon development), and GO:0010817 (regulation of hormone levels). Genes in MEgreen were enriched in GO:0071417 (cellular response to organonitrogen compound), GO:0017038 (protein import), GO:0042391 (regulation of membrane potential), GO:0005773 (vacuole), GO:0018105 (peptidyl-serine phosphorylation). Genes in MEturquoise were mainly enriched in GO:0016604 (nuclear body), GO:0006397 (mRNA processing), GO:0006403 (RNA localization), GO:0006753 (nucleoside phosphate metabolic process), and GO:0005635 (nuclear envelope). For the top 20 clusters enriched in each module, please refer to Supplementary Table 1.

The KEGG pathway analysis showed that genes in MEblue were mainly enriched in hsa04010 (MAPK signaling pathway), hsa01522 (Endocrine resistance), and hsa05414 (Dilated cardiomyopathy), and genes in MEgreen were mainly enriched in hsa03450 (Non-homologous end-joining). No significantly enriched pathways were found for MEturquoise.

### Visualization of the PPI Network and Hub Genes

**Figure 5:**
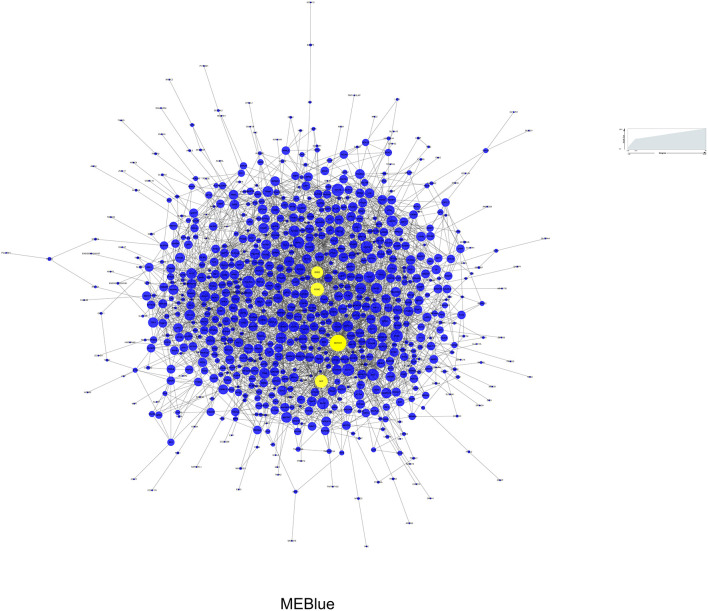
Protein-protein interaction network of MEBlue for BD. Yellow node represents the most important protein of the network and the related gene is defined as hub gene. Node size represents the degree of connectivity.

Among the 3 modules, MEblue (*r* = 0.39, *P* = 4e-04) and MEgreen (*r* = −0.39, *P* = 5e-04) had the strongest correlations with BD, and the number of genes in MEblue was greater than that in MEgreen. Therefore, we selected the MEblue module for the subsequent analysis (32). The PPI network analysis was performed on the MEblue module using the STRING database. As a result, 853 nodes and 2,687 edges were established in the module and the PPI enrichment *P*-value was 1.0e-12. The interactions of proteins in MEblue were selected and converted into a network, which was visualized with Cytoscape, as shown in Figure 5. Each term is represented by a circular node, and its cluster identity is represented by its color. The top 10 hub genes in the PPI network were calculated by CytoHubba (28). As a result, four genes (*NOTCH1, POMC, NGF*, and *DRD2*), which overlapped among the five methods, were deemed hub genes. Then we analyzed the gene expression levels of these hub genes, and found that, compared with controls, the expression level of *NOTCH1* (*t*_75_ = 2.159, *P* = 0.034), *NGF* (*t*_75_ = 3.183, *P* = 0.002), and *POMC* (*t*_75_ = 3.791, *P* = 3e-04) presented significant up-regulation in BD (Supplementary Figure 4).

### Validation of Gene Function, Annotation Enrichment, and Hub Genes

In an independent dataset, GSE12649, we carried out the same analyses as above to validate our results. WGCNA revealed seven coexpression modules in this dataset. Among these modules, MEbrown (including 460 genes) showed a significant positive correlation (*r* = 0.27, *P* = 0.04) with BD (Supplementary Table 2). In this module, we then validated the top five GO functional enrichment results of each module and four KEGG pathways we observed previously (Supplementary Table 3). The following GO enrichment results appeared in the clusters of MEbrown: GO:0071417 (cellular response to organonitrogen compound), GO:0030029 (actin filament-based process), GO:0003012 (muscle system process), GO:0061564 (axon development), GO:0010817 (regulation of hormone levels), Go:0042391 (regulation of membrane potential). Among the four important KEGG pathways, only the hsa05414 (Dilated cardiomyopathy) was verified in the MEbrown. Finally, among the four hub genes of the main results, *NOTCH1* was verified as a hub gene in this validation dataset (Supplementary Table 4).

## Discussion

In this study, we applied WGCNA to the BD gene expression profiles GSE5388 and GSE538 and identified 10 modules on the top 5,000 genes from 77 samples. Three out of 10 modules were significantly associated with BD. Functional enrichment and the PPI network of these BD-related modules were explored with Metascape and the STRING database. We then identified four genes, *NOTCH1, POMC, NGF*, and *DRD2*, as hub genes underlying BD. In a further validation analysis using a separate dataset (GSE12649), we validated several biological processes and pathways (such as actin filament-based process, axon development, hormone level regulation) and *NOTCH1* as a hub gene of BD.

To understand the genetic mechanisms of BD, various studies have been carried out, adopting various genetic techniques such as microarray, single-cell sequencing, RNA-sequencing, and GWAS. The microarray datasets we analyzed were contributed by Ryan and colleagues; their results suggest that BD is associated with the dysregulation of the ubiquitin pathway and synaptic genes in orbitofrontal cortex (16). As we mentioned in the Introduction, given the complex and multifactorial nature of BD genetic mechanisms, here we investigated the potential alterations of the interconnection between genes in BD using WGCNA (33). We identified three critical modules in the main analyses, which are associated with various biological processes and pathways, such as muscle system processes, biosynthesis of carbohydrate derivatives, actin filament formation, axon development, hormone level regulation, MAPK signaling pathway, and endocrine resistance. Some of these findings were confirmed in the validation analysis in a separate dataset, such as actin filament-based process, axon development, hormone level regulation. Previous studies have revealed alterations of gene sets mediating synaptic transmission and nervous system development in BD based on microarray technology (16), pathway-based analyses (34) or prioritized gene framework (35) of the genome-wide association datasets. Other studies have highlighted many other pathways, showing convergence on neuroplasticity (5–7). Our findings of actin filament-based process and axon development are in line with consistent observations of neuroplasticity alterations in BD and provide evidence for potential frontal structural plasticity abnormalities in BD.

The most significant KEGG pathways associated with BD were the MAPK signaling pathway and endocrine resistance. A GWAS study has shown that one of the KEGG pathways involved in BD was the MAPK signaling pathway (36), which is involved in mediating entrainment of the circadian system (37). Many circadian genes have been associated with BD (38). The MAPK change in the intracellular signal cascade may be caused by the immune imbalance in BD (39). Endocrine resistance refers to resistance to endocrine therapy agents, such as selective estrogen receptor modulators (e.g., tamoxifen). Some studies have found that tamoxifen was effective against manic episodes (40) and our study lends further support to the potential role of endocrine resistance in BD pathogenesis. Nevertheless, these two pathways were not confirmed in the validation dataset. Instead, both primary and validation datasets revealed that BD-related modules are mainly enriched in has05414 (Dilated cardiomyopathy). Future studies are warranted to examine whether this pathway is involved in the pathophysiology of BD or a by-product of long-term mood stabilizer treatment.

The Notch signaling pathway plays critical roles in neural development and brain homeostasis and is involved in neuronal migration, early differentiation, memory formation, and synaptic plasticity (41). The Notch receptor contains four members (Notch1, Notch2, Notch3, and Notch4), and their expression patterns in the forebrain are as follows: *NOTCH1* in neurons, astrocytes, precursors, ependymal cells, and endothelium; *NOTCH2* in neurons and precursors; *NOTCH3* in precursors; *NOTCH4* in the endothelium (42). The Notch pathway is closely associated with the pathological mechanism of BD (43, 44). For example, the *NOTCH4* gene expression in peripheral blood cells has been found to be upregulated in BD (45). The *NOTCH3* mutation has also been reported in BD, which however was not consistent across studies (46, 47). A recent WGCNA study with relatively small samples (17 BD vs. 19 controls) from the prefrontal cortex reported *NOTCH2* as *one* of the 30 hub genes in BD (48). Our results highlight *NOTCH1* as a hub gene for BD in both primary and validation datasets. While these results together suggest the involvement of Notch pathway in BD, the inconsistent findings across studies may arise from differences in tissue types, analysis methods, and sample sizes. Intriguingly, the *NOTCH1* signaling could be activated by valproic acid (49), a commonly used mood stabilizer, which suggests that NOTCH1 may serve as a potential treatment target for BD.

Our main results also revealed *POMC, NGF*, and *DRD2* as hub genes for BD. Proopiomelanocortin (POMC)-derived peptides are involved in the regulation of energy homeostasis, learning, memory, inflammation, and immune modulation (50, 51). The significantly increased levels of the critical pituitary hormones POMC indicated dysfunction of the HPA axis of BD (52, 53). *POMC* is also one of the shared genes between mood disorder and cardiometabolic diseases (54). In the central nervous system, nerve growth factor (NGF) play key roles in neuroprotection and neural repair (55). Based on published GWASs and candidate gene studies, the *NGF* gene might be a useful biological marker for the manic state and early detection of conversion from significant depression to BD (56–58). Finally, the *DRD2* gene encodes the D2 subtype of the dopamine receptor. Several studies have shown that the *DRD2* gene is associated with BD and that polymorphism in *DRD2* may play a role in BD development (59–61). However, these candidate genes were not observed in the validation dataset and their involvement in BD warrants further scrutiny.

## Limitations

Our study has some limitations. First, the small sample size for WGCNA may affect the robustness of the observed results. Sample sizes in our main and validation datasets met the minimum requirements for WGCNA (i.e., larger than 15 samples per group) (62), but may be not large enough to detect modules with smaller effect sizes. Future studies with larger sample sizes are needed to validate our findings. Second, compared to RNA-sequencing, the microarray data is limited by the probes pre-defined by the manufacturer and is not sensitive to low-abundance genes. As the RNA-sequencing data storage and analysis become increasingly available to researchers, future studies have the opportunities to reveal other potential BD-related genetic alterations. Third, the brain tissue we analyzed contained multiple cell types, which may have different gene expression profiles. For instance, the *NOTCH1* seems to have higher expression levels in glia cells than in neurons [Supplementary Figure 5, based on the single-cell RNA sequence of 466 cells in the healthy human brain in a public scRNA-seq database scRNASeqDB (63, 64)]. Our study may fail to detect BD-related changes in specific cell types; it is also unclear whether our results were driven by one or some cell types. Future investigation is warranted to reveal the cell-type-specific alterations associated with BD. Finally, this was a preliminary study by using public data, and the results need to be further validated with molecular biology experiments. Although some essential genes and pathways were verified in another dataset (GSE12649), the reliability of our study was still insufficient and the findings should be interpreted with caution.

## Conclusions

In summary, we performed WGCNA in independent gene expression datasets and highlighted *NOTCH1* as one candidate gene of BD and the involvement of several biological processes such as actin filament-based process and axon development, which might be targets for BD diagnosis and treatment. These results provide new perspectives for understanding BD pathogenesis and invite further investigations and validations on the candidate genes and biological processes/pathways we observed.

## Data Availability Statement

Publicly available datasets were analyzed in this study. This data can be found here: https://www.ncbi.nlm.nih.gov/geo/.

## Author Contributions

Z-QZ, W-WW, J-TL, and T-MS designed the study and wrote the manuscript. Z-QZ, J-DC, G-YZ, and J-YL prepared and analyzed the data. Y-KW, YZ, and Y-AS prepared and reviewed the study. All authors contributed to the article and approved the submitted version.

## Conflict of Interest

The authors declare that the research was conducted in the absence of any commercial or financial relationships that could be construed as a potential conflict of interest.
