# Supplementary material for: Weighted Gene Coexpression Network Analysis Reveals Essential Genes and Pathways in Bipolar Disorder
Source: Front Psychiatry. 2021 Mar 17;12:553305. doi: 10.3389/fpsyt.2021.553305 (PMC8010671; doi:10.3389/fpsyt.2021.553305)
Supplement: Supplementary file 1 [file Data_Sheet_1.docx]

***Supplementary Material***

Weighted gene coexpression network analysis reveals essential genes and pathways in bipolar disorder

Zhen-Qing Zhang, Wei-Wei Wu, Jin-Dong Chen, Guang-Yin Zhang, Jing-Yu Lin, Yan-Kun Wu, Yu Zhang, Yun-Ai Su, Ji-Tao Li, Tian-Mei Si

**Legends of Supplementary Figure and Table**

**Supplementary Figure 1.** Quality control plots for GSE5388 and GSE5389. Outliers were determined based on standardized network connectivity z-scores. Available covariates and potential confounding factors balanced groups, including sex, age, pH, PMI, RNAdeg, and batch. PMI: post-mortem interval; RNAdeg: RNA degradation.

**Supplementary Figure 2.** Quality control plots after GSE5388 and GSE5389 merged. The batch effect between GSE5388 and GSE5389 was shown. Multidimensional scaling plots and Mega−Analysis show sample clustering by the first two expression principal components.

**Supplementary Figure 3.** Sample clustering to detect outliers. 5,000 genes were selected by WGCNA and hclust function, shared with the highest variable expression values. All the 77 samples were clarified into one cluster.

**Supplementary Figure 4.** Gene expression levels of potential hub genes in the BD and control groups.

**Supplementary Figure 5.** The expression levels of *NOTCH1* in different cell types in the healthy human brains.

**Supplementary Table 1.** Top 20 clusters in each module with their representative enriched terms. "Count" is the number of genes in the user-provided lists with membership in the given ontology term. Log10(q)" is the Multi-test adjusted p-value in log base 10.

**Supplementary Table 2.** Module-Trait relationships in the validation dataset GSE12649.

**Supplementary Table 3.** The validated clusters in MEbrown with their enriched terms.

**Supplementary Table 4.** The top ten hub genes in the PPI network of MEbrown in the dataset GSE12649 by CytoHubba using five methods.


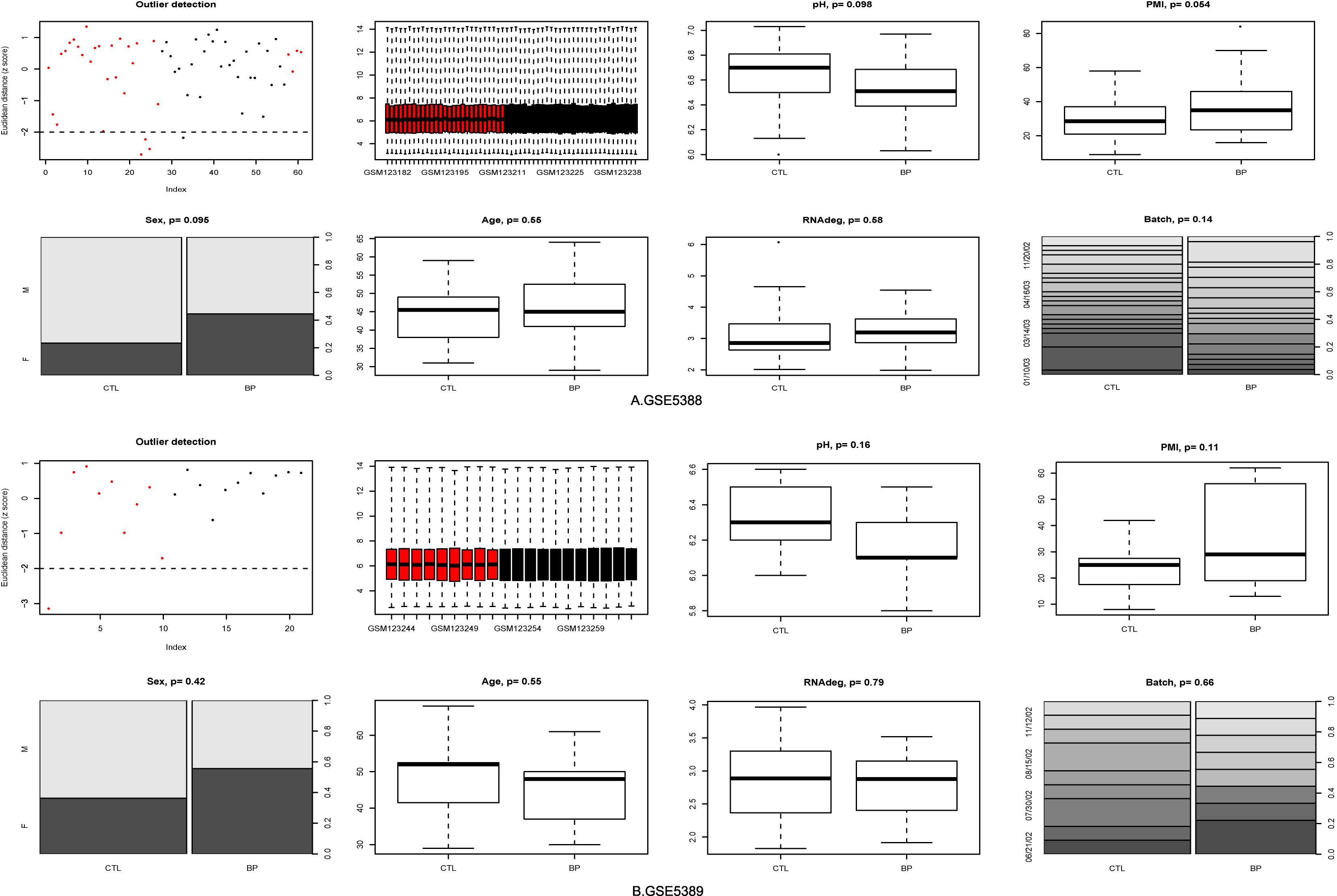


**Supplementary Figure 1.** Quality control plots for GSE5388 and GSE5389. Outliers were determined based on standardized network connectivity z-scores. Available covariates and potential confounding factors balanced groups, including sex, age, pH, PMI, RNAdeg, and batch. PMI: post-mortem interval; RNAdeg: RNA degradation.


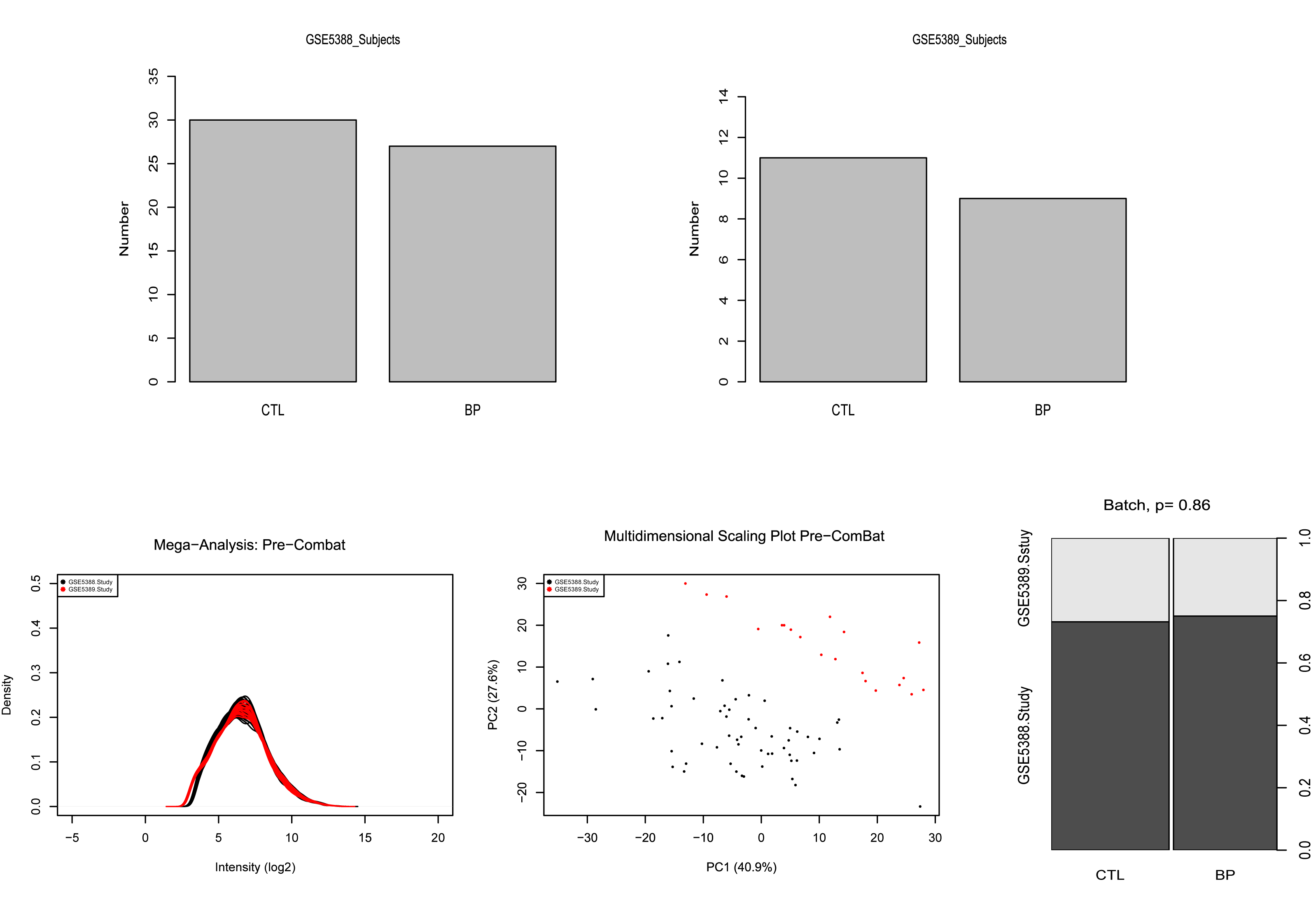


**Supplementary Figure 2.** Quality control plots after GSE5388 and GSE5389 merged. The batch effect between GSE5388 and GSE5389 was shown. Multidimensional scaling plots and Mega−Analysis show sample clustering by the first two expression principal components.


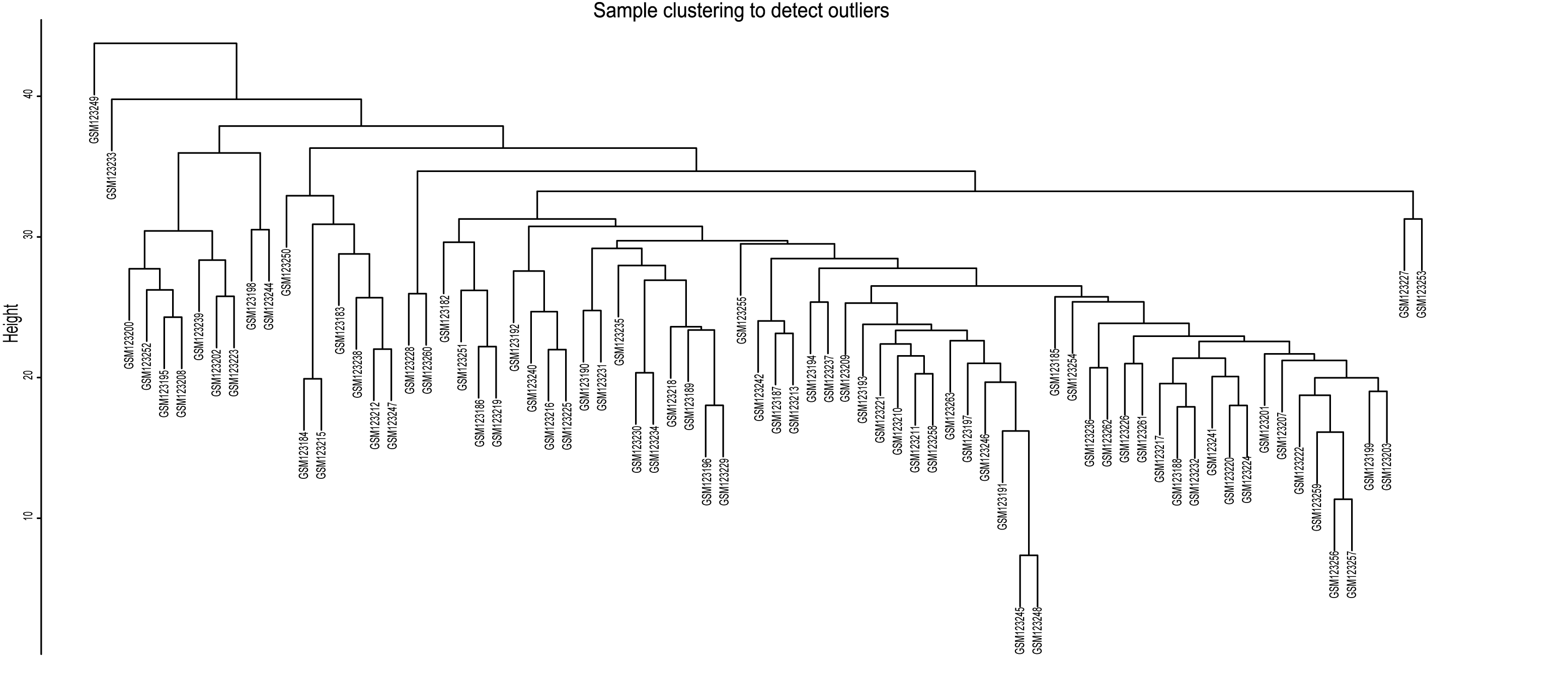


**Supplementary Figure 3.** Sample clustering to detect outliers. 5,000 genes were selected by WGCNA and hclust function, shared with the highest variable expression values. All the 77 samples were clarified into one cluster.

otento
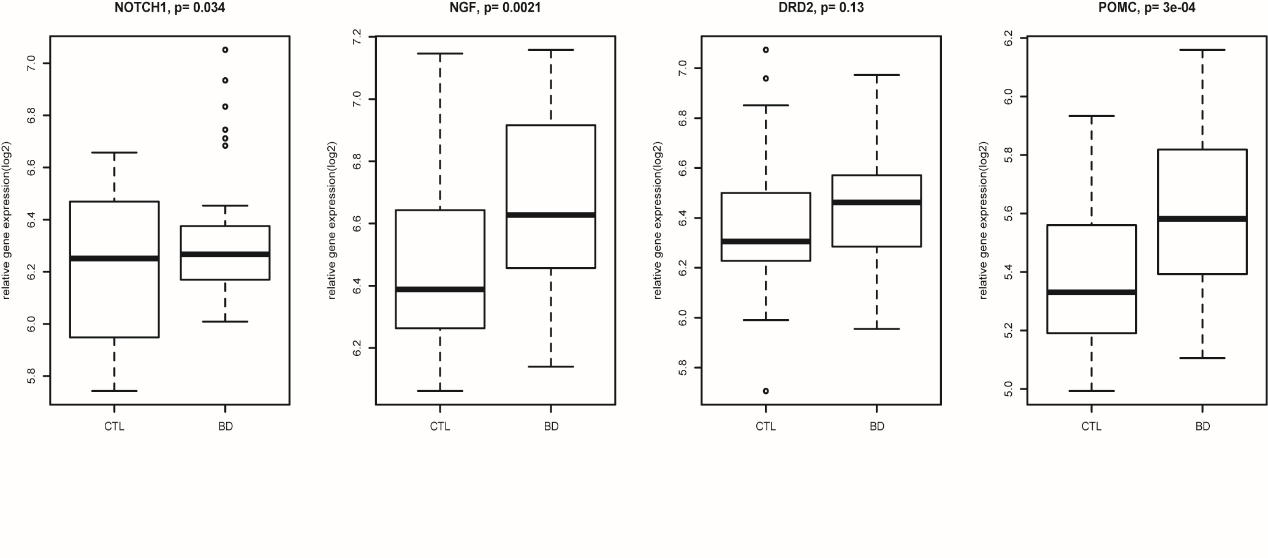
**Supplementary Figure 4.** Gene expression levels of potential hub genes in BD and control groups.


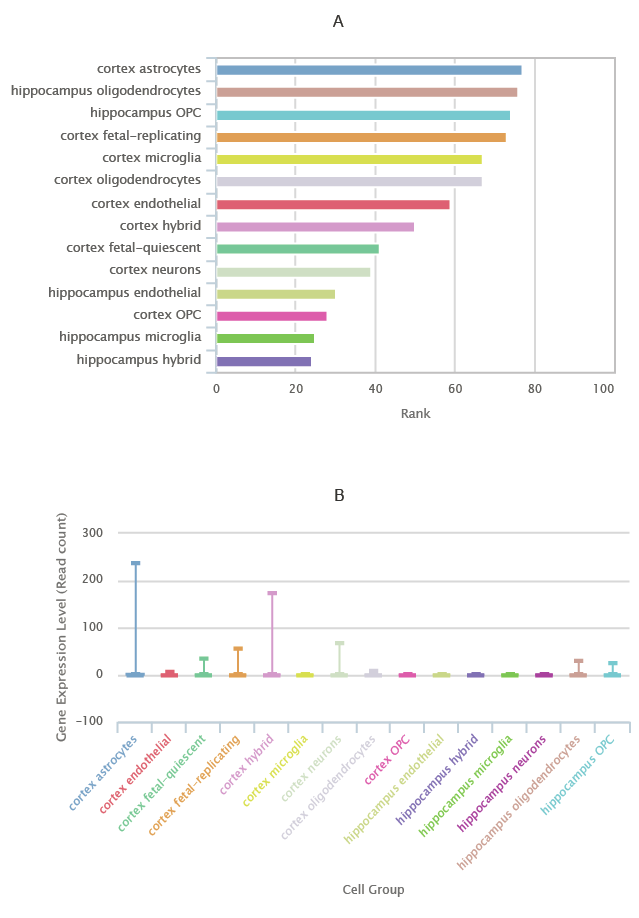


**Supplementary Figure 5.** The expression levels of *NOTCH1* in different cell types in the healthy human brains, based on the single-cell RNA sequence of 466 cells in a public scRNA-seq database scRNASeqDB. (A) Rank of *NOTCH1* expression in GSE67835. (B) *NOTCH1* expression in each cell group.

**Supplementary Table 1.** Top 20 clusters in each module with their representative enriched terms. "Count" is the number of genes in the user-provided lists with membership in the given ontology term. Log10(q)" is the Multi-test adjusted p-value in log base 10.

| Module GO Category Description Count Log10(q) |
| --- |
| MEblue GO:0003012 BP muscle system process 44 -4.62  MEblue GO:1901137 BP carbohydrate derivative biosynthetic process 61 -4.62  MEblue GO:0030029 BP actin filament-based process 60 -4.62  MEblue GO:0061564 BP axon development 45 -4.28  MEblue GO:0010817 BP regulation of hormone levels 46 -4.26  MEblue GO:0007517 BP muscle organ development 38 -3.87  MEblue GO:0003206 BP cardiac chamber morphogenesis 19 -3.71  MEblue GO:0043062 BP extracellular structure organization 37 -3.45  MEblue GO:0034330 BP cell junction organization 29 -3.40  MEblue GO:0008610 BP lipid biosynthetic process 51 -3.17  MEblue hsa04010 KEGG MAPK signaling pathway 26 -3.12  MEblue hsa01522 KEGG Endocrine resistance 15 -3.12  MEblue GO:0050890 BP cognition 28 -3.08  MEblue GO:0050804 BP modulation of chemical synaptic transmission 36 -3.02  MEblue GO:0007420 BP brain development 51 -2.98  MEblue hsa05414 KEGG Dilated cardiomyopathy 14 -2.89  MEblue GO:0008202 BP steroid metabolic process 29 -2.61  MEblue GO:0007568 BP aging 28 -2.51  MEblue GO:1903827 BP regulation of cellular protein localization 39 -2.49  MEblue GO:0071363 BP cellular response to growth factor stimulus 48 -2.47  MEgreen GO:0071417 BP cellular response to organonitrogen compound 24 -3.16  MEgreen GO:0017038 BP protein import 13 -2.76  MEgreen GO:0042391 BP regulation of membrane potential 19 -2.76  MEgreen GO:0005773 CC vacuole 26 -2.76  MEgreen GO:0018105 BP peptidyl-serine phosphorylation 15 -2.71  MEgreen GO:0098662 BP inorganic cation transmembrane transport 25 -2.71  MEgreen GO:0035384 BP thioester biosynthetic process 7 -2.54  MEgreen GO:0050808 BP synapse organization 17 -2.37  MEgreen GO:0031625 MF ubiquitin protein ligase binding 14 -2.33  MEgreen GO:0006820 BP anion transport 21 -2.28  MEgreen hsa03450 KEGG Non-homologous end-joining 4 -2.21  MEgreen GO:0019904 MF protein domain specific binding 22 -2.21  MEgreen GO:0030425 CC dendrite 20 -2.12  MEgreen GO:0060078 BP regulation of postsynaptic membrane potential 9 -1.98  MEgreen GO:0070585 BP protein localization to mitochondrion 9 -1.98  MEgreen GO:0000139 CC Golgi membrane 22 -1.98  MEgreen GO:0060589 MF nucleoside-triphosphatase regulator activity 14 -1.98  MEgreen GO:1905114 BP cell surface receptor signaling pathway 19 -1.74  involved in cell-cell signaling  MEgreen GO:0005759 CC mitochondrial matrix 16 -1.72  MEgreen GO:0048193 BP Golgi vesicle transport 14 -1.69  MEturquoise GO:0016604 CC nuclear body 97 -9.16  MEturquoise GO:0006397 BP mRNA processing 73 -8.08  MEturquoise GO:0006403 BP RNA localization 43 -8.08  MEturquoise GO:0006753 BP nucleoside phosphate metabolic process 86 -7.86  MEturquoise GO:0005635 CC nuclear envelope 64 -7.53  MEturquoise GO:0051656 BP establishment of organelle localization 67 -7.36  MEturquoise GO:0098984 CC neuron to neuron synapse 52 -6.91  MEturquoise GO:0080135 BP regulation of cellular response to stress 85 -6.64  MEturquoise GO:0044089 BP positive regulation of cellular component biogenesis 67 -6.27  MEturquoise GO:1903311 BP regulation of mRNA metabolic process 47 -5.89  MEturquoise GO:0090407 BP organophosphate biosynthetic process 78 -5.89  MEturquoise GO:0050803 BP regulation of synapse structure or activity 38 -5.89  MEturquoise GO:0043632 BP modification-dependent macromolecule catabolic process 73 -5.71  MEturquoise GO:1990234 CC transferase complex 83 -5.53  MEturquoise GO:0097447 CC dendritic tree 70 -5.28  MEturquoise GO:0048471 CC perinuclear region of cytoplasm 75 -4.64  MEturquoise GO:0009266 BP response to temperature stimulus 36 -4.63  MEturquoise GO:0070628 MF proteasome binding 9 -4.61  MEturquoise GO:0007610 BP behavior 65 -4.56  MEturquoise GO:0051129 BP negative regulation of cellular component organization 75 -4.24 |

BP: biological process; CC: cellular component; MF: molecular function.

**Supplementary Table 2.** Module-Trait relationships in the validation dataset GSE12649.

| Module | Trait | Person Correlation Value | *P*-value |
| --- | --- | --- | --- |
| MEblue  MEbrown  MEgreen  MEgrey  MEred  MEturquoise  MEyellow | Disease  Disease  Disease  Disease  Disease  Disease  Disease | 0.15  0.27  0.13  0.04  0.10  -0.09  -0.03 | 0.25  0.04  0.32  0.75  0.43  0.52  0.82 |

**Supplementary Table 3.** The validated clusters in MEbrown with their enriched terms.

| Module | Go | Category | Description | Count | Log10(q) |
| --- | --- | --- | --- | --- | --- |
| MEbrown  MEbrown  MEbrown  MEbrown  MEbrown  MEbrown  MEbrown | GO:0071417  GO:0030029  GO:0003012  GO:0061564  GO:0010817  GO:0042391  hsa05414 | BP  BP  BP  BP  BP  BP  KEGG Pathway | cellular response to organonitrogen compound  actin filament-based process  muscle system process  axon development  regulation of hormone levels  regulation of membrane potential  Dilated cardiomyopathy | 35  36  24  26  24  16  24 | -6.2  -4.2  -3.4  -3.4  -2.5  -0.92  -1 |

BP: biological process.

**Supplementary Table 4.** The top ten hub genes in the PPI network of MEbrown in the dataset GSE12649 by CytoHubba using five methods.

| Rank | MNC | | Degree | | Closeness | | EPC | | Radiality | |
| --- | --- | --- | --- | --- | --- | --- | --- | --- | --- | --- |
|  | Name | Score | Name | Score | Name | Score | Name | Score | Name | Score |
| 1  2  3  4  5  6  7  8  9  10 | EGFR  NOTCH1  FGF2  DECR1  APOE  GFAP  CD44  VWF  AGT  TLR4 | 72  55  49  43  40  38  37  34  30  25 | EGFR  NOTCH1  FGF2  DECR1  GFAP  APOE  CD44  VWF  AGT  SOX9 | 79  57  51  44  41  41  41  36  32  27 | EGFR  NOTCH1  FGF2  DECR1  GFAP  CD44  APOE  VWF  AGT  ITPKB | 218.58333  197.83333  196.83333  189.95  189.31667  187.1  185.65  183.85  180.45  176.08333 | EGFR  NOTCH1  FGF2  CD44  GFAP  APOE  VWF  ITPKB  AGT  DECR1 | 103.165  101.626  100.718  98.685  97.52  96.921  94.886  91.209  90.701  90.286 | EGFR  FGF2  NOTCH1  DECR1  GFAP  CD44  VWF  APOE  AGT  ITPKB | 6.88601  6.68803  6.66773  6.6195  6.61443  6.57889  6.56113  6.55605  6.52813  6.51036 |

MNC: maximum neighborhood component; EPC: edge percolated component.
